# Factors driving co-occurrence of Schistosoma mansoni and S. haematobium at the micro-geographical level

**DOI:** 10.1186/2049-3258-73-S1-P26

**Published:** 2015-09-17

**Authors:** Lynn Meurs, Moustapha Mbow, Maria Yazdanbakhsh, Katja Polman

**Affiliations:** 1 Institute of Tropical Medicine, Antwerp, Belgium; 2 Aristide Le Dantec Teaching Hospital, Dakar, Senegal; 3 Leiden University Medical Center, Leiden, the Netherlands

## Brief introduction

Several studies have shown that the two major human *Schistosoma* species, *Schistosoma mansoni* and *Schistosoma haematobium*, co-exist in the same individuals at the micro-geographical level. However, the drivers of this co-occurrence are still largely unknown. Here, we studied whether coexposure and/or host immune responses might explain this phenomenon.

## Methods and materials

A multidisciplinary study was conducted in two neighboring rural communities in northern Senegal, where *S. mansoni* and *S. haematobium* are co-endemic (n=857). Kato-Katz and urine filtration were used for microscopic detection of the respective *Schistosoma* species. Households were located using handheld differential global positioning system in the largest community (n=599), and the Kulldorff's scan statistic was used to detect spatial clusters of infection. *Schistosoma*-specific cytokine responses (IL-10, IL-5, IFN-γ, TNF-a, and IL-2) were assessed in 72h whole blood culture supernatants (n=200), and analyzed by the multivariate technique nonmetric multidimensional scaling (nMDS).

## Results

Classical epidemiological analyses showed that the two parasites were more likely to co-occur in the same individuals (p<0.001). Moreover, co-infected subjects had significantly higher infection levels than their mono-infected counterparts (p<0.001; adjusted for age and gender). In contrast, micro-geographical analyses revealed a very focal spatial distribution with *S. mansoni* clustering in one (p=0.002) and *S. haematobium* infections in another section (p=0.023) of the community. nMDS analysis of the cytokine data indicated that the characteristic modified Th2 response was most pronounced in co-infected subjects.

## Conclusions

The divergent geographical distribution of *S. mansoni* and *S. haematobium* in this community could not explain why the two infections cluster in the same individuals. This implies that co-infection is not driven by coexposure, but by within-host interactions. Cytokine profiles suggested that co-occurrence of the two species may be due to host immunological factors and/or parasite-induced immunomodulation. However, other factors may also play a role.

